# Association between maternal psychological adversity and lung function in South African infants: A birth cohort study

**DOI:** 10.1002/ppul.24532

**Published:** 2019-09-30

**Authors:** Symon M. Kariuki, Diane M. Gray, Charles R. J. C. Newton, Aneesa Vanker, Rae P. MacGinty, Nastassja Koen, Whitney Barnett, Lori Chibnik, Karestan C. Koenen, Dan J. Stein, Heather J. Zar

**Affiliations:** ^1^ Department of Clinical Research (Neurosciences) KEMRI‐Wellcome Trust Research Programme Kilifi Kenya; ^2^ Department of Psychiatry University of Oxford Oxford England; ^3^ Department of Paediatrics and Child Health, Red Cross War Memorial Children's Hospital University of Cape Town Cape Town South Africa; ^4^ Department of Paediatrics and Child Health South African Medical Research Council (SAMRC) Unit on Child and Adolescent Health Cape Town South Africa; ^5^ Department of Psychiatry & Neuroscience Institute University of Cape Town Cape Town South Africa; ^6^ Department of Psychiatry and Mental Health South African Medical Research Council (SAMRC) Unit on Anxiety and Stress Disorders Cape Town South Africa; ^7^ Harvard TH Chan School of Public Health Harvard University Boston Massachusetts

**Keywords:** Africa, children, infant lung function, psychological adversity, risk factors

## Abstract

**Objective:**

The association of perinatal psychological adversity (ie, stressors and distress) with infant lung function (ILF) and development is not well studied in Africa and elsewhere. We determined the association between maternal perinatal psychological adversity and ILF in African infants.

**Design:**

Prospective longitudinal follow up of the Drakenstein Child Health Study birth cohort.

**Participants:**

Seven hundred and sixty‐two infants aged 6 to 10 weeks and 485 infants who had data for both maternal perinatal psychological adversity and ILF (measured at 6 to 10 weeks and 12 months).

**Methods:**

The main analyses were based on cross‐sectional measures of ILF at each assessment (6 to 10 weeks or 12 months), using generalized linear models, and then on the panel‐data of both longitudinal ILF assessments, using generalised estimating equations, that allowed specification of the within‐group correlation structure.

**Results:**

Prenatal intimate partner violence (IPV) exposure was associated with reduced respiratory resistance at 6 to 10 weeks (beta coefficient [*β*] = −.131, *P* = .023); postnatal IPV with reduced ratio of time to peak tidal expiratory flow over total expiratory time (t_PTEF_/t_E_) at 12 months (*β* = −.206, *P* = .016); and prenatal depression with lower respiratory rate at 6 to 10 weeks (*β* = −.044, *P* = .032) and at 12 months (*β* = −.053, *P* = .021). Longitudinal analysis found an association of prenatal IPV with reduced t_PTEF_/t_E_ (*β* = −.052, *P* < .0001); postnatal IPV with decreased functional residual capacity (FRC; *β* = −.086, *P* < .0001); prenatal posttraumatic stress disorder with increased FRC (*β* = .017, *P* < .0001); prenatal depression with increased FRC (*β* = .026, *P* < .0001) and postnatal depression with increased FRC (*β* = .021, *P* < .0001).

**Conclusion:**

Screening for psychological adversity and understanding the mechanisms involved may help identify children at risk of altered lung development and inform approaches to treatment.

## 1 INTRODUCTION

Prenatal and postnatal psychological adversity (ie, stressors and distress) is common worldwide, especially in low and middle income countries, where depression, for example, is reported in 8% to 30% of mothers.^1^ Similarly, respiratory diseases are major causes of childhood morbidity and mortality, particularly in African children.^2^ Epidemiological studies show significant associations between prenatal and postnatal maternal psychosocial adversity and respiratory diseases in infants particularly wheezing,^3^ but these studies are from high‐income countries and are limited by ascertainment of respiratory disease and heterogeneity of definitions use. Recently we reported an association between maternal postnatal psychological adversity, intimate partner violence (IPV), and wheezing in infants in the Drakenstein Child Health Study, an African birth cohort study,^4^ but impact on infant lung function (ILF) measures has not been examined in this setting.

Lung function can provide an objective measure of lung health that can be longitudinally measured. Recently we have shown that lung function can be reliably measured in infants at 6 weeks and at 12 months of age, providing robust data on lung health in African infants.^5^, ^6^ Several hypotheses about underlying biology have been put forward to explain the association between maternal psychological adversity and ILF. Stressor induced changes in maternofetal immune functioning during the prenatal period, and a consequent proinflammatory state,^7^ is one hypothesized pathway for influencing respiratory disease susceptibility in infants.

In addition to our work on the association of maternal psychological adversity with wheezing,^4^ we have found that respiratory illnesses may be comorbid with behavioural and emotional problems in young children.^8^ Importantly, however, any association between psychological adversity and ILF can be confounded by other factors such as benzene exposure and height‐for‐age z‐scores (which were associated with ILF in a previous related study).^6^, ^9^ Socioeconomic status should also be addressed since it can contribute both to ILF and to psychological adversity.^10^ Similarly, maternal HIV and antiretroviral drugs may influence a child's growth and development,^11^ as it is thought antiretroviral drugs could be passed to the unborn child and influence child development.^12^

Identifying the shared risk factors for maternal psychological adversity and ILF is important as these factors may modify or moderate the association between the two conditions. The Drakenstein Child Health Study^13^, ^14^ in a periurban area of South Africa (SA), offers a unique opportunity to understand the relationship between maternal psychological adversity and ILF using a birth cohort design and empirical measures. The aim of this nested study was to determine the association between maternal perinatal psychological adversity and ILF (measured at 6‐10 weeks and at 12 months).

## 2 METHODS AND MATERIALS

### 2.1 Population and site

Mother‐infant dyads were enrolled into the Drakenstein Child Health Study, to investigate the epidemiology, aetiology, and determinants of child health with longitudinal follow‐up of children until they are at least 5‐years of age.^13^, ^14^ The study was conducted in a periurban, low socioeconomic community, about 60 km from Cape Town in SA. Pregnant mothers were enrolled in their second trimester through the local primary health care clinics. Psychological adversity was assessed before birth and soon after birth,^13^ environmental exposures, and clinical measures were obtained before birth,^14^ and ILF was measured at 6 to 10 weeks and 1 year of age.^6^, ^9^

### 2.2 Assessment of maternal psychosocial adversity

Prenatal and postnatal psychological adversity included in this analysis were: (a) IPV, (b) posttraumatic stress disorder (PTSD), and (c) depressive symptoms. Prenatal assessments for psychological adversity were done before birth while postnatal assessments were evaluated either at 6 to 10 weeks after birth.

#### 2.2.1 Intimate partner violence

IPV was assessed with an IPV tool, which was piloted in the local South African population and found to be reliable when used in this population.^13^, ^15^ The tool comprises three sub‐scales as well as an overall IPV score based on frequency of abuse: (emotional abuse [four questions], physical abuse [four questions], and sexual abuse [three questions]), both of which are scored on a Likert scale of 1‐4, representing the frequency an abuse happened (1 = never, 2 = once, 3 = few, and 4 = many). Lifetime or past 12 months exposure for each subscale is generated. Emotional abuse was assessed by asking the mother about being insulted or made to feel bad, being belittled or humiliated in public, and being purposefully scared or intimidated. Physical abuse included history of being beaten (eg, by fist), pushed by force, injured (eg, burns), or threatened by weapons. Sexual abuse was assessed by asking about history of forced sexual intercourse when unwilling or forced into sexual activity that was humiliating. The responses for each question and scale were summed up to generate a continuous total score based on the frequency of abuse.

#### 2.2.2 Posttraumatic stress disorder

PTSD was assessed with the Modified PTSD Symptom Scale (MPSS) which has 18 questions, with responses organised into 4‐likert scale (0‐3).^16^ The scales measure the frequency of PTSD symptoms, with 0 representing absence of symptoms, 1 present once, 2 presence of two to four symptoms, and 3 presence of 5 or more symptoms.^13^ The final 18th item assesses for duration of symptoms, with response options including less than 1 month; 1 to 3 months; 3 months to 1 year; and more than 1 year. The PTSD scores were then organised into “no exposure,” “suspected exposure,” and “definite exposure i.e. suspected exposure to PTSD” as previously described.^13^, ^15^ Previous studies have used the MPSS due to its good diagnostic validity for PTSD and good psychometric properties including concurrent validity.

#### 2.2.3 Perinatal depression

The Edinburgh Postnatal Depression Rating Scale (EPDS), is a 10‐item self‐report measure of depressive symptoms in the past 1 week.^17^ Items are scored on a frequency scale, ranging from 0 to 3. A continuous score was obtained by summing the individual items; with the lowest scores representing absent or nonsevere depressive symptoms and vice versa.^13^ It has been piloted and used in SA and found to possess good psychometric properties.^18^

### 2.3 Assessment of ILF

ILF was tested at 6 to 10 weeks and 1 year of age, with the child in quiet natural sleep and included tidal breathing, multiple breath washout measures, and the forced oscillation technique as previously described.^6^, ^9^ Measures of ILF were first validated on normative data and found to be reliable before their application in this cohort.^5^ The following ILF parameters were obtained: tidal volume (mL), ratio of time to peak tidal expiratory flow over total expiratory time (t_PTEF_/t_E_), respiratory rate (per minute), functional residual capacity (FRC, mL), respiratory system resistance (cmH_2_O·L·s^−1^), and compliance (cmH_2_O·mL^−1^).

All lung function measurements conformed to American Thoracic Society/European Respiratory Society guidelines, as previously published.^5^ Tidal breathing and flow volume loops (TBFVL) and multiple breath washout (MBW), performed using 4% SF6 as a tracer gas, were collected using the Exhalyzer D with ultrasonic flow meter (Ecomedics AG, Duernten, Switzerland) and mean measures calculated with acquisition and analysis software (Wbreath v3.28.0, Ndd Medizintechnik AG). The forced oscillation technique (FOT) measurement was made with purpose‐built equipment (University of Szeged, Hungary). Composite medium frequency signal (8‐48 Hz) was delivered to the infants via a wave‐tube through a facemask covering the mouth and nose.

### 2.4 Assessment of environmental and medical confounding factors

Socioeconomic status was based on a composite score for education, employment, income, assets and market access, and organised into four quartiles ranging from high to low. Maternal smoking during pregnancy was based on maternal urine cotinine (IMMULITE 1000 Nicotine Metabolite Kit; Siemens Medical Solutions Diagnostics, Glyn, Rhonwy, UK), with levels more than 500 ng/mL considered active smokers, 10 to 500 passive, and less than 10 nonsmokers.^19^ Benzene was considered present if household levels more than 5 μg/m^3^.^20^ Maternal alcohol was assessed with the Alcohol, Smoking and Substance Involvement Screening Test self‐reported questionnaires. Maternal respiratory illnesses were considered present if there was history of asthma, chronic cough, or recurrent wheeze in previous 12 months and/or low forced expiratory volume.^6^, ^9^

### 2.5 Ethics approval

The study was approved by the Ethics Committee of the Faculty of Health Sciences, University of Cape Town, by Stellenbosch University and the Western Cape Provincial Research committee. Written informed consent was obtained from parents and is renewed annually.

### 2.6 Data analysis

All the analyses were done using R statistical software (version 3.1.0 [2014‐04‐10], http://www.r-project.org) and Stata version 13 (Stata Corp, TX). Some psychosocial adversity variables (IPV and depression) were continuous and skewed and thus logarithmic (natural) transformation was performed to try to reduce the skewness of the residuals from regression models.

The main analyses were based on cross‐sectional measures of ILF at each assessment (at 6‐10 weeks and at 12 months), using gamma regression, a type of generalized linear model (GLM) that assumes a gamma distribution for the outcome. This is more appropriate than a Gaussian distribution since our outcomes can only take on positive values, are slightly skewed and are log‐transformed. In the cross‐sectional analysis, all children at each assessment were included to allow comparisons in future studies since those missed at 12 months assessments may reinter the study in future follow‐ups. For our panel‐data of two ILF assessments, we used generalised estimating equations (GEEs), that allowed specification of the within‐group correlation structure, using only those children included in both 6 to 10 weeks and 12 months; the response variables were measures of ILF, while assessments of maternal perinatal psychological adversity were the explanatory variables. As with the GLM models, we specified a gamma distribution for our outcomes and an exchangeable correlation structure. In these GLM and GEE analyses, we first constructed unadjusted models, then adjusted models (accounting for potential confounders in particular, sex, socioeconomic status, population group, exposure to benzene, perinatal complications and maternal age, height‐for‐age z scores, respiratory illness, and HIV), and finally tested if there was interaction in the adjusted model between psychological adversity and maternal health behaviour variables (smoking, alcohol consumption, and breastfeeding). Height‐for‐age z scores added into the GLM and GEE models were available for both assessments (at 6‐12 weeks and at 12 months assessments).

Other comparisons between two groups were done with the Wilcoxon signed‐rank test for non‐normal continuous scores and Pearson's *χ* ^2^ tests for frequency distributions.

## 3 RESULTS

### 3.1 General description

ILF data were obtained at 6 to 10 weeks in 762 children in whom measures of maternal psychological adversity were also administered; these were followed to obtain environmental and medical history as well as psychological adversity exposures. Of the 762 children, ILF was assessed again at 12 months in 485 (64%), representing those who had data at the two time points that is at baseline (6‐10 weeks) and at 12 months (Figure S1) and were therefore included in the current analysis.

### 3.2 Distribution of sociodemographic and other environmental/perinatal factors

Males comprised 391 of 762 (51%) of the children with ILF tests at 6 to 10 weeks, and 238 of 485 (49%) of those with ILF tests at 12 months, and the proportion of males did not differ between the two follow‐up periods.

The proportion of children born to a black African mother was similar for the first ILF tests at 6 to 10 weeks (393/762 [52%]) and follow‐up period at 12 months (230/485 [47%]; *P* = .157). The distribution of children exposed to benzene during the first ILF tests at 6 to 10 weeks (250/472 [53%]) and follow‐up at 12 months was similar (156/310 [50%]; *P* = .469). Of the 762 children who had ILF tests at 6 to 10 weeks, 94 (12%) were born premature, compared to 62 (13%) who were found to have been born premature at 12 months (*P* = .815). The proportion of HIV exposure among these 762 and 485 children was 19% and 19%, respectively. The distribution of other environmental/perinatal factors is shown in Table 1.

**Table 1:** Description of sociodemographic and environmental, infant lung function, and psychological adversity variables

	ILF test at 6‐10 weeks (N = 762)	ILF followed up at 12 months (N = 485)^a^	*P* ^b^
Child's male sex	391 (51%)	238 (49%)	.440
Maternal smoking (cotinine)			
Passive	149/486 (31%)	94/310 (30%)	.920
Active	237/486 (49%)	162/310 (52%)	.336
Gestation age (GA)			
Large (weight >90th %centile GA)	35 (5%)	23 (5%)	.903
Normal (GA not large or small)	531 (70%)	334 (69%)	.759
Small weight <10th %centile GA	196 (26%)	128 (26%)	.792
Prematurity (delivery <37 weeks)	94 (12%)	62 (13%)	.815
Birth season			
Autumn	188 (25%)	137 (28%)	.160
Spring	171 (22%)	98 20%)	.349
Summer	216 (28%)	150 (31%)	.329
Winter	187 (24%)	100 (21%)	.108
Abnormal delivery mode			
Assisted breech	4/759 (1%)	2 (<1%)	.775
Elective CS	54/759 (7%)	38 (8%)	.635
Emergency CS	97/759 (13%)	49 (10%)	.152
Mother breastfeeds child	670/759 (88%)	427 (88%)	.901
Socioeconomic status			
High	187 (25%)	118 (24%)	.932
Moderate‐high	192 (25%)	126 (26%)	.757
Low‐moderate	190 (25%)	130 (27%)	.461
Low	193 (25%)	111 (23%)	.327
Black African ancestry of mother	393 (52%)	230 (47%)	.152
Maternal alcohol use in pregnancy	157/447 (35%)	101/297 (34%)	.754
Presence of benzene in household	250/472 (53%)	156/310 (50%)	.469
Maternal age in years: mean (IQR)	27 (6)	27 (23‐31)	
Maternal respiratory illness	36 (5%)	25 (5%)	.731
Maternal HIV	146 (19%)	92 (19%)	.933
Maternal psychological adversity variables			
Intimate partner violence			
Prenatal, median (IQR)	14.0 (12.0‐17.0)	14 (12‐18)	.596
Postnatal, median (IQR)	12.0 (12.0‐16.0)	12.0 (12.0‐16.0)	.594
Prenatal posttraumatic stress disorders			
Suspected	94/688 (13.7%)	62/436 (14.2%)	.813
Definite	92/688 (13.4%)	53/436 (12.1%)	.526
Postnatal posttraumatic stress disorders			
Suspected	5/99 (5.0%)	4/66 (6.1)	.760
Definite	4/99 (4.0%)	2/66 (3.0)	.736
Depression scores			
Prenatal, median IQR	9.0 (6.0‐13.0)	9.0 (6.0‐13.0)	.758
Postnatal, median IQR	8.0 (5.0‐11.0)	8.0 (5.0‐11.0)	.771
Infant lung function variables			
Tidal volume (mL), median (Q1‐Q3)	34.6 (30.8‐38.6)	91.5 (82.6‐101.5)	<.001
Ratio of time to peak tidal expiratory flow over total expiratory time, median (IQR)	38.1 (29.6‐46.1)	28.3 (22.6‐36.1)	<.001
Respiratory rate (breaths per minutes), median (IQR)	47.0 (40.5‐54.7)	29 (25‐33)	<.001
Functional respiratory capacity (mL), median (IQR)	75.4 (66.1‐86.6)	201.2 (171.0‐226.7)	<.001
Respiratory system resistance (cmH_2_O·L·s^−1^), median (IQR)	44.7 (37.2‐55.0)	NA	
Respiratory system compliance (cmH_2_O·mL^−1^)	96.1 (73‐122)	NA	

IQR, interquartile range; ILF, infant lung function; NA, not applicable; Q, quartile.

^a^ Includes only those with psychological data at both baseline and follow up.

^b^ Two sample comparison of proportions and means.

### 3.3 Distribution of prenatal and postnatal psychological adversity factors

The IPV scores were higher prenatally than postnatally (*P* = .0012; Table 1). However, there were no differences in prenatal and postnatal IPV measures between the two ILF test periods (*P* = .596 and .594, respectively). The frequency of definite exposure to trauma i.e. suspected exposure to PTSD was higher prenatally than postnatally (*P* = .007; Table 1). However, there were no differences in frequency of prenatal and postnatal PTSD between the two ILF test periods (*P* = .526 and .736, respectively). A similar pattern was observed for prenatal and postnatal EPDS scores over the two ILF test periods (Table 1).

### 3.4 Distribution of ILF measures

Six ILF tests were included in this analysis, for which four were measured at both time points and two, namely respiratory system resistance and compliance, were only available at first ILF tests measured at 6 to 10 weeks (Table 1). Lung function changed as expected with somatic growth between 6 to 10 weeks and 12 months.

### 3.5 Cross‐sectional association between psychological adversity measures and ILF measured at 6 to 10 weeks and 12 months

In multivariable models with ILF measures at 6 to 10 weeks as response variables, prenatal IPV was associated with decreased FRC (beta coefficient [*β*] = −.005 [95% confidence interval {CI} = 0.011, 0.000]) and with decreased respiratory resistance (*β* = −.131 [95% CI = −0.244, −0.018]; Table 2). In similar models, prenatal depression was associated with increased FRC (*β* = .002 [95% CI = 0.000, 0.005]) and with lower respiratory rate (*β* = −.044 [95% CI = −0.085, −0.037]). There were no significant univariable associations between psychological adversity measures and ILF measures at 6 to 10 weeks (Tables S1‐S5).

**Table 2:** Adjusted cross‐sectional association between psychological adversity measures and lung function at 6 to 10 weeks and 12 months

Psychological adversity factors	FRC		Respiratory rate, bpm		Resistance cmH_2_O·L·s^−1^		t_PTEF_/t_E_	
	Coef (95% CI)	*P*	Coef (95% CI)	*P*	Coef (95% CI)	*P*	Coef (95% CI)	*P*
ILF measures at 6‐10 weeks								
Prenatal IPV	**−.005 (−0.011, 0.000)**	**.036**	−.057 (−0.147, 0.032)	.210	**−.131 (−0.244, −0.018)**	**0.023**	−.068 (−0.182, 0.045)	.241
Prenatal depression	**.002 (0.000, 0.005)**	**.048**	**−.044 (−0.085, −0.037)**	**.032**	.044 (−0.051, 0.060)	0.869	.008 (−0.057, 0.074)	.802
ILF measures at 12 months								
Postnatal IPV	−.003 (−0.001, 0.004)	.409	−.065 (−0.213, 0.082)	.388	FN	FN	**−.206 (−0.374, −0.037)**	**.016**
Prenatal depression	**.002 (0.000, 0.005)**	**.054**	**−.053 (−0.098, −0.008)**	**.021**	FN	FN	−.013 (−0.084, 0.058)	.716

*Note*: Associations highlighted in bold are statistically significant. Models adjusted for sex, socioeconomic status, race, exposure to benzene, height‐for‐age z scores, perinatal complications and maternal age, respiratory illness, or HIV. Bold values represent associations that reached a P‐value of <0.05. PTSD had few numbers to run any associations, so results not shown.

Abbreviations: bpm, breaths per minute; CI, confidence interval; FN, too few numbers or no observations to run the model; FRC, functional residual capacity; IPV, intimate partner violence, NB, no biological plausibility for postnatal psychosocial adversity to influence infant lung function at 6‐10 wk as both were measured around the same time; PTSD, posttraumatic stress disorder; t_PTEF_/t_E,_ ratio of time to peak tidal expiratory flow over total expiratory time.

^a^Assumption of no departure from linear trend for postnatal PTSD because of small samples.

In multivariable models with ILF measures at 12 months, postnatal IPV was associated with reduced t_PTEF_/t_E_ (*β* = −.206 [95% CI = −0.374, −0.037]) only (Table 2). Postnatal depression was associated with increased FRC (*β* = .002 [95% CI = 0.000, 0.005]) and with reduced respiratory rate (*β* = −.053 [95% CI = −0.098, −0.008]). The univariable analysis results for the association between psychological adversity measures and ILF measures at 12 months are shown in Tables S1‐S5.

### 3.6 Longitudinal associations between psychological adversity measures and ILF measures from 6‐10 weeks through 12 months

In longitudinal models, which accounted for within‐group correlations in ILF measures change over 1 year, definite prenatal PTSD was associated with all 4 measures: positively with FRC (*β* = .045 [95% CI = 0.035, 0.055]) and tidal volume (*β* = .026 [95% CI = 0.004, 0.048]) and negatively with respiratory rate (*β* = −.027 [95% CI = −0.053, −0.001]) and t_PTEF_/t_E_ (*β* = −.078 [95% CI = −0.104, −0.052]; Table 3). In similar models prenatal PTSD (with assumptions for no linear departure for trend) was associated with three measures: FRC (*β* = .017 [95% CI = 0.010, 0.023]), tidal volume (*β* = .011 [95% CI = 0.010, 0.011]) and t_PTEF_/t_E_ (*β* = −.034 [95% CI = −0.040,−0.027]). Prenatal IPV was negatively associated with FRC (*β* = −.037 [95% CI = −0.074, −0.001]) and t_PTEF_/t_E_ (*β* = −.052 [95% CI = −0.079,−0.026]). Similarly postnatal IPV was negatively associated with fractional residual capacity (*β* = −.086 [95% CI = −0.108, −0.064]) and t_PTEF_/t_E_ (*β* = −.200 [95% CI = −0.223, −0.176]). Increased FRC was predicted by both prenatal depression (*β* = .026 [95% CI = 0.023, 0.028]) and postnatal depression (*β* = .021 [95% CI = 0.017, 0.025]).

**Table 3:** Longitudinal association between psychological adversity measures and infant lung function measures accounting for within group correlations from 6 to 10 weeks through 12 months

	Functional residual capacity		Tidal volume		Respiratory rate		t_PTEF_/t_E_	
	Adjusted *β* coefficient (95% CI)	*P*	Adjusted *β* coefficient (95% CI)	*P*	Adjusted β coefficient (95% CI)	*P*	Adjusted *β* coefficient (95% CI)	*P*
Prenatal IPV	**−.037 (−0.074, −0.001)**	**.043**	.006 (−0.079, 0.092)	.884	−.054 (−0.133, 0.024)	.179	**−.052 (−0.079, −0.026)**	**<.0001**
Postnatal IPV	**−.086 (−0.108, −0.064)**	**<.0001**	.008 (−0.080, 0.098)	.845	−.030 (−0.072, 0.10)	.145	**−.200 (−0.223, −0.176)**	**<.0001**
Prenatal PTSD exposure								
None	Ref		Ref		Ref		Ref	
Suspected exposure	−.009 (−0.078, 0.059)	.789	.002 (−0.048, 0.052)	.934	−.026 (−0.083, 0.030)	.367	−.006 (−0.033, 0.019)	.599
Definite exposure	**.045 (0.035, 0.055)**	**<.0001**	**.026 (0.004, 0.048)**	**.018**	**−.027 (−0.053, −0.001)**	**.038**	**−.078 (−0.104, −0.052)**	**<.0001**
Prenatal PTSD exposure^a^	**.017 (0.010, 0.023)**	**<.0001**	**.011 (0.010, 0.011)**	**<.0001**	−.015 (−0.036, 0.004)	.125	**−.034 (−0.040, −0.027)**	**<.0001**
Prenatal depression	**.026 (0.023, 0.028)**	**<.0001**	.002 (−0.013, 0.019)	.731	−.019 (−0.089, 0.051)	.595	−.002 (−0.026, 0.022)	.855
Postnatal depression	**.021 (0.017, 0.025)**	**<.0001**	.002 (−0.003, 0.007)	.462	−.004 (−0.024, 0.014)	.635	.036 (−0.011, 0.084)	.138

*Note*: Models adjusted for sex, socioeconomic status, race, exposure to benzene, height‐for‐age z scores, perinatal complications and maternal age, respiratory illness, or HIV. Bold values represent associations that reached a P‐value of <0.05. There were no results for the associations with postnatal PTSD exposure because of few observations.

Abbreviations: CI, confidence interval; IPV, intimate partner violence, PTSD, posttraumatic stress disorder. tPTEF/tE, ratio of time to peak tidal expiratory flow over total expiratory time.

^a^ Assumption of no departure from linear trend for postnatal PTSD because of small samples.

### 3.7 Interaction of psychological adversity with maternal smoking, alcohol consumption and breastfeeding in the adjusted models

There was evidence for interaction between psychological adversity factors and maternal smoking, alcohol consumption, and breastfeeding in explaining the risk for ILF measures (Table S6). The risk of tidal volume, FRC, and respiratory rate were the ILF measures that were most saliently explained by the interaction between these three maternal variables (maternal smoking, alcohol consumption, and breastfeeding) and prenatal IPV or postnatal IPV.

## 4 DISCUSSION

This longitudinal study demonstrates that perinatal psychological adversity may influence measures of infant lung development and function throughout infancy. Psychological adversity was associated with impaired lung volumes, flow and respiratory rate, suggesting that they may have an effect on both structural lung development and control of breathing. There is also evidence that psychological adversity factors interact with maternal smoking, alcohol consumption and breastfeeding to influence ILF and development, either synergistically or antagonistically. These associations differed according to the type of ILF or psychological adversity, and according to follow‐up period (at 6‐10 weeks or 12 months, or when both assessment periods are considered).

The frequency or median distribution of all psychological adversity measures was similar between 6 and 10 weeks and 12 months, suggesting that there was no bias related to attrition between the two periods. This observation was supported by similar distribution of sociodemographic characteristics for the child (eg, sex) and the mother (socioeconomic status and ethnicity) between the two periods. The persistence of these maternal psychological adversity factors between prenatal and postnatal periods (eg, depression was 20% prenatally and 12% postnatally) is important,^21^ this chronicity of exposure may be an important consideration as these factors may continue to affect a child's development after birth and throughout early childhood. Changes in ILF measures observed between 6 and 10 weeks and at 12 months follow‐ups as expected are due to maturation changes with age.

We found significant associations between psychological adversity and ILF and development, after adjusting for potential confounders. In multivariable cross‐sectional associations at 6 to 10 weeks, only prenatal IPV, and prenatal depression were associated with ILF and development, notably FRC and respiratory rate. FRC, like tidal volume may represent structural lung development while respiratory rate may reflect control of breathing,^6^, ^9^ suggesting that both lung structure and breathing control may be affected by the two psychological adversity factors. However, tidal volume may also be affected by breathing control and similarly, respiratory rate by lung volume.

Similar psychological adversity factors, but only those measured postnatally, were associated with ILF at 12 months, for example postnatal IPV with t_PTEF_/t_E_ and postnatal depression with fractional residual capacity and respiratory rate, again implying that both structural and functional aspects of the respiratory system are affected. Previous paediatric studies have associated childhood psychological adversity with symptoms of asthma,^22^ which may be related to the altered respiratory function measured in our study. In both time periods, there were fewer significant associations of ILF with PTSD than there were with IPV or depression, which may be due to fewer observations for PTSD. Other ILF measures particularly respiratory system resistance and compliance did not show significant associations in the cross‐sectional analysis at 6 to 10 weeks. Given recent evidence of significant associations between maternal depression at any year with wheezing at 3 years in USA and SA,^3^, ^4^ the impact of psychological adversity on respiratory illness as well as on lung function should be examined in future studies.

The longitudinal associations of psychological adversity and lung function across two time periods were interesting in that suspected exposure to PTSD emerged as an important predictor of most ILF measures across the two study follow‐up periods, with a positive association for tidal volume and fractional residual capacity, and negative association with respiratory rate and t_PTEF_/t_E,_ suggesting differential effects on both lung development and breathing functions. The negative association between ILF and respiratory rate is perhaps because tidal volume was higher; the two ILF measures are important in determining minute ventilation. Unlike PTSD and depression, which had positive associations with FRC, IPV consistently had negative associations with FRC, again suggesting differential impact for each psychological adversity factor on ILF and development. We previously demonstrated the association of these ILF measures with respiratory illnesses.^4^

Interaction analysis from this study may help clarify how maternal factors such as alcohol consumption and smoking, identified as risk factors for infant lung development and function in a related study,^6^, ^9^ contribute to lung function and development. For example, IPV showed more significant interaction with maternal in influencing ILF and development, than did other psychological factors. It is possible that mothers who experience violence in pregnancy resort to alcohol consumption and smoking and vice versa, with both possibilities resulting in detrimental consequences on the developing lung. Additionally, mothers with high levels of psychological adversity may not exclusively breastfeed their children, contributing to alterations in development.^23^ While each of these factors may individually contribute to poor lung function and development, this study shows that they may also interact with one another either synergistically or antagonistically to determine the risk of lung function and development.

Children of mothers with psychosocial adversity may inherit limited passive immunity that predisposes them to respiratory illnesses, consequently compromising their lung development and function. The immunity of the child's mother may be perturbed by a direct effect of psychological adversity on the nerves terminating on immune tissue or indirectly through engaging in habits (eg, smoking) that suppress the immune system.^24^ Maternal psychological adversity may also impact secretion of cortisol, adrenaline and noradrenaline, all of which may cross the placenta and influence development of vital organs including lungs.^25^ Specifically, this may result in adverse hypothalamic–pituitary–adrenal development in the child that in turn may influence maturation and differentiation of vital fetal organs including the lungs and brain.^26^ Socioeconomic status—which may moderate psychological adversity—may also affect child development through social causation and social selection, with those who are socially disadvantaged have less access to health and nutrition.^10^ It is thought that alcohol consumption, which is influenced by psychological adversity, directly affects the respiratory system function for example reduced protein surfactant, but more evidence is needed in human studies. HIV infection, which cause psychological adversity, is neurotropic,^11^ and neurotoxic antiretroviral drugs may be passed to the unborn child, thus also influencing child development.^27^

Epigenetic pathways have also been proposed; maternal psychosocial adversity may modify child gene activity without necessarily altering gene sequence.^28^ This may, in turn, alter gene expression in the brain or lungs impairing the physiological functions of these organs. A review by Monk et al^29^ proposed that maternal psychological adversity may result in methylation of infant DNA or altered gene expression in the placenta or infant lungs, although further empirical studies are needed to support this observation. In addition, evidence shows that there are shared susceptibility genes that are expressed in both the brain and respiratory system.^30^, ^31^ Shared genes are worth investigation in future studies, for example the beta gene coding for pituitary adenylate cyclase–activating polypeptide receptor 1 (ADCYAP1R1: a susceptibility gene for PTSD and anxiety), was associated with reduced bronchodilator response in children with asthma from Puerto Rico.^32^ More importantly, genetic and environment factors may interact to contribute both to psychosocial adversity, and to ILF and development, which should be taken into consideration in future epidemiological studies.

## 5 STRENGTHS AND LIMITATIONS

The strength of this study is the use of a large birth cohort, which allows one to observe health outcomes following a risk factor exposure. Use of multiple comprehensive measures improves the sensitivity of identifying psychological adversity and ILF and development, thus producing reliable associations. The statistical analysis included initial checks to decide on the best model fit for these data and potential confounders were accounted for in the final association models. Measures of ILF were first validated before being applied on this cohort.^5^ The limitations are inability to account for residual confounding of factors not measured in this study. Reporting of historical events for example depressive symptoms may be subject to recall bias, while social disirability bias may influence responses to alcohol intake or IPV, for instance. Some ILF tests have poor reliability (sensitivity and specificity) in detecting lung function and development.

## 6 CONCLUSIONS

This study has demonstrated that there is an association between psychological adversity and ILF, which remained after accounting for potential confounding factors, and that ILF and development may be influenced by an interaction between psychosocial adversity and maternal habits such as smoking and alcohol consumption. Psychological adversity may affect both structural development and control of respiratory function. Thus, psychological adversity should be screened for and addressed in expectant or new mothers. Our observation that prenatal psychological adversity affected lung function measured at 6 to 10 weeks underlines the importance of identifying and managing psychosocial adversity early in pregnancy.^33^ It is possible that there are shared genes or epigenetic modifications may affect both psychosocial adversity and ILF; this is a promising direction for future studies. As the follow‐up period was short in our settings, there are plans to continue with long‐term measurement of lung function measures in children born to mothers with exposure to psychological adversity examining potential effects on other aspects of child's growth and neurodevelopment.

## CONFLICT OF INTERESTS

The authors declare that there are no conflict of interests.

## Supporting information

Supporting information

Supporting information
