# Supplementary material for: Association between maternal psychological adversity and lung function in South African infants: A birth cohort study
Source: Pediatr Pulmonol. 2019 Sep 30;55(1):236–44. doi: 10.1002/ppul.24532 (PMC7154702; doi:10.1002/ppul.24532)
Supplement: Supplementary file 1 — Supporting information [file PPUL-55-236-s001.docx]

**Supplementary file**

**Supplementary table 1. Cross-sectional associations between** **psychosocial stress measures and functional residual capacity**

| **Psychosocial stress factors** | At baseline (6-10 weeks) | | | | At follow-up (12 months) | | | |
| --- | --- | --- | --- | --- | --- | --- | --- | --- |
|  | Crude models (95%CI) | | Adjusted models (95%CI) | | Crude models (95%CI) | | Adjusted models (95%CI) | |
|  | **β Coefficient (95%CI)** | p-value | **β Coefficient (95%CI)** | p-value | **β Coefficient (95%CI)** | p-value | **β Coefficient (95%CI)** | p-value |
| **Prenatal IPV** | -0.002  (-0.006,0.001) | 0.292 | **-0.005**  **(-0.011,0.000)** | **0.036** | -0.002  (-0.007,0.002) | 0.268 | -0.003  (-0.009,0.003) | 0.320 |
| **Postnatal IPV** | NA | NA | NA | NA | **-0.006**  **(-0.012,0.000)** | **0.050** | -0.003  (-0.001,0.004) | 0.409 |
| **Prenatal PTSD** |  |  |  |  |  |  |  |  |
| None | Ref |  | Ref |  | Ref |  | Ref |  |
| Suspected exposure | 0.001  (-0.002,0.004) | 0.445 | 0.001  (-0.003,0.006) | 0.485 | 0.000  (-0.004,0.003) | 0.873 | 0.001  (-0.03,0.007) | 0.548 |
| Definite exposure | 0.001  (-0.002,0.005) | 0.390 | 0.001  (-0.004,0.007) | 0.687 | 0.001  (-0.003,0.006) | 0.634 | 0.003  (-0.003,0.009) | 0.311 |
| **Prenatal PTSD^a^ exposure** | 0.000  (0.000,0.002) | 0.316 | 0.000  (-0.001,0.003) | 0.564 | 0.000  (-0.001,0.002) | 0.705 | 0.001  (-0.001,0.004) | 0.268 |
| **Postnatal PTSD^a^ exposure** | NA | NA | NA | NA | 0.001  (-0.006,0.009) | 0.730 | FN | FN |
| **Prenatal depression** | -0.000  (-0.002,0.001) | 0.853 | **0.002**  **0.000,0.005)** | **0.048** | 0.000  (-0.001,0.002) | 0.765 | **0.002**  **(0.000,0.005)** | **0.054** |
| **Postnatal depression** | NA | NA | NA | NA | 0.000  (-0.002,0.002) | 0.945 | 0.001  (-0.001,0.004) | 0.285 |

^a^Assumption of no departure from linear trend for postnatal ASD because of small samples. IPV: intimate partner violence, PTSD: post-traumatic stress disorder. NA: no biological plausibility for postnatal psycho social stress to influence infant lung function at 6-10 weeks as both were measured around the same time. FN: few numbers to run the model. Models adjusted for sex, socioeconomic status, race, exposure to benzene, perinatal complications and maternal age, respiratory illness or HIV

**Supplementary table 2. Cross-sectional associations between** **psychosocial stress measures and tidal volume**

| **Psychosocial stress factors** | At baseline (6-10 weeks) | | | | At follow-up (12 months) | | | |
| --- | --- | --- | --- | --- | --- | --- | --- | --- |
|  | Crude models (95%CI) | | Adjusted models (95%CI) | | Crude models (95%CI) | | Adjusted models (95%CI) | |
|  | **β Coefficient (95%CI)** | p-value | **β Coefficient (95%CI)** | p-value | **β Coefficient (95%CI)** | p-value | **β Coefficient (95%CI)** | p-value |
| **Prenatal IPV** | -0.099  (-0.055,0.036) | 0.680 | 0.011  (-0.046,0.068) | 0.707 | 0.006  (-0.048,0.060) | 0.828 | 0.038  (-0.031,0.108) | 0.279 |
| **Postnatal IPV** | NA | NA | NA | NA | 0.045  (-0.029,0.12) | 0.231 | 0.070  (-0.014,0.155) | 0.103 |
| **Prenatal PTSD exposure** |  |  |  |  |  |  |  |  |
| None | Ref |  | Ref |  | Ref |  | Ref |  |
| Suspected exposure | 0.026  (-0.011,0.064) | 0.166 | 0.013  (-0.030,0.057 | 0.557 | 0.012  (-0.029,0.053) | 0.696 | 0.029  (-0.028,0.086) | 0.319 |
| Definite exposure | 0.010  (-0.028,0.048) | 0.601 | -0.008  (-0.067,0.050) | 0.778 | -0.010  (-0.061,0.040) | 0.696 | 0.016  (-0.049,0.083) | 0.620 |
| **Prenatal PTSD^a^ exposure** | 0.008  (-0.009,0.026) | 0.361 | -0.001  (-0.028,0.025) | 0.927 | -0.001  (-0.025, 0.021) | 0.870 | 0.011  (-0.019,0.042) | 0.461 |
| **Postnatal PTSD^a^ exposure** | NA | NA | NA | NA | -0.002  (-0.098,0.092) | 0.951 | FN | FN |
| **Prenatal depression** | -0.001  (-0.021,0.018) | 0.876 | 0.001  (-0.002,0.004) | 0.572 | 0.004  (-0.018,0.026) | 0.719 | 0.002  (-0.001,0.005) | 0.206 |
| **Postnatal depression** | NA | NA | NA | NA | 0.000  (-0.026,0.026) | 0.986 | 0.017  (-0.014,0.049) | 0.276 |

^a^Assumption of no departure from linear trend for postnatal ASD because of small samples. IPV: intimate partner violence, PTSD: post-traumatic stress disorder. NA: no biological plausibility for postnatal psycho social stress to influence infant lung function at 6-10 weeks as both were measured around the same time. FN: few numbers to run the model. Models adjusted for sex, socioeconomic status, race, exposure to benzene, perinatal complications and maternal age, respiratory illness or HIV

**Supplementary table 3. Cross-sectional associations between** **psychosocial stress measures and respiratory rate**

| **Psychosocial stress factors** | At baseline (6-10 weeks) | | | | At follow-up (12 months) | | | |
| --- | --- | --- | --- | --- | --- | --- | --- | --- |
|  | Crude models (95%CI) | | Adjusted models (95%CI) | | Crude models (95%CI) | | Adjusted models (95%CI) | |
|  | **β Coefficient (95%CI)** | **p-value** | **β Coefficient (95%CI)** | **p-value** | **β Coefficient (95%CI)** | **p-value** | **β Coefficient (95%CI)** | **p-value** |
| **Prenatal IPV** | -0.35  (-0.092,0.020) | 0.215 | -0.057  (-0.147,0.032) | 0.210 | -0.048  (-0.116,0.019) | 0.164 | -0.086  (-0.187,0.014) | 0.092 |
| **Postnatal IPV** | NA | NA | NA | NA | -0.069  (-0.179,0.006) | 0.069 | -0.065  (-0.213,0.082) | 0.388 |
| **Prenatal PTSD exposure** |  |  |  |  |  |  |  |  |
| None | Ref |  | Ref |  | Ref |  | Ref |  |
| Suspected exposure | -0.009  (-0.055,0.035) | 0.672 | -0.032  (-0.099,0.035) | 0.347 | -0.011  (-0.065,0.042) | 0.677 | -0.053  (-0.133,0.025) | 0.186 |
| Definite exposure | -0.008  (-0.059,0.042) | 0.745 | -0.010  (-0.087,0.067) | 0.796 | 0.010  (-0.059,0.080) | 0.771 | -0.034  (-0.118,0.048) | 0.409 |
| **Prenatal PTSD^a^ exposure** | -0.005  (-0.029,0.018) | 0.675 | -0.009  (-0.045,0.026) | 0.594 | 0.002  (-0.029,0.033) | 0.894 | -0.023  (-0.062,0.016) | 0.247 |
| **Postnatal PTSD^a^ exposure** | NA | NA | NA | NA | 0.056  (-0.028,0.141) | 0.193 | FN | FN |
| **Prenatal depression** | -0.005  (-0.034,0.023) | 0.699 | **-0.044**  **(-0.085,-0.037)** | **0.032** | -0.026  (-0.058,0.006) | 0.121 | **-0.053**  **(-0.098,-0.008)** | **0.021** |
| **Postnatal depression** | NA | NA | NA | NA | 0.013  (-0.025,0.052) | 0.496 | -0.007  (-0.058,0.044) | 0.789 |

^a^Assumption of no departure from linear trend for postnatal ASD because of small samples. IPV: intimate partner violence, PTSD: post-traumatic stress disorder. NA: no biological plausibility for postnatal psycho social stress to influence infant lung function at 6-10 weeks as both were measured around the same time. FN: few numbers to run the model. Models adjusted for sex, socioeconomic status, race, exposure to benzene, perinatal complications and maternal age, respiratory illness or HIV

**Supplementary table 4. Cross-sectional associations between** **psychosocial stress measures and ratio of time to peak tidal expiratory flow over total expiratory time (**t_PTEF_/t_E_**)**

| **Psychosocial stress factors** | **At baseline (6-10 weeks)** | | | | **At follow-up (12 months)** | | | |
| --- | --- | --- | --- | --- | --- | --- | --- | --- |
|  | Crude **models (95%CI)** | | **Adjusted models (95%CI)** | | Crude **models (95%CI)** | | **Adjusted models (95%CI)** | |
|  | **β Coefficient (95%CI)** | **p-value** | **β Coefficient (95%CI)** | **p-value** | **β Coefficient (95%CI)** | **p-value** | **β Coefficient (95%CI)** | **p-value** |
| **Prenatal IPV** | -0.062  (-0.146,0.021) | 0.143 | -0.068  (-0.182,0.045) | 0.241 | **-0.107**  **(-0.214,0.000)** | **0.052** | -0.061  (-0.195,0.072) | 0.368 |
| **Postnatal IPV** | NA | NA | NA | NA | **-0.209**  **(-0.360,-0.059)** | **0.006** | **-0.206**  **(-0.374,-0.037)** | **0.016** |
| **Prenatal PTSD exposure** |  |  |  |  |  |  |  |  |
| None | Ref |  | Ref |  | Ref |  | Ref |  |
| Suspected exposure | 0.004  (-0.063,0.072) | 0.895 | 0.000  (-0.099,0.101) | 0.985 | 0.000  (-0.081,0.082) | 0.985 | -0.019  (-0.134,0.096) | 0.746 |
| Definite exposure | 0.021  (-0.051,0.093) | 0.567 | -0.071  (-0.169,0.025) | 0.149 | 0.022  (-0.064,0.109) | 0.617 | -0.049  (-0.015,0.059) | 0.371 |
| **Prenatal PTSD^a^ exposure** | 0.009  (-0.024,0.043) | 0.581 | -0.029  (-0.076,0.017) | 0.218 | 0.009  (-0.032,0.050) | 0.663 | -0.024  (-0.076,0.028) | 0.373 |
| **Postnatal PTSD^a^ exposure** | NA | NA | NA | NA | 0.13 (-0.007,0.274) | 0.064 | FN | FN |
| **Prenatal depression** | 0.008  (-0.035,0.052) | 0.713 | 0.008  (-0.057,0.074) | 0.802 | 0.000  (-0.055,0.055) | 0.999 | -0.013  (-0.084, 0.058) | 0.716 |
| **Postnatal depression** | NA | NA | NA | NA | 0.030  (-0.029,0.090) | 0.321 | 0.006  (-0.069,0.083) | 0.862 |

^a^Assumption of no departure from linear trend for postnatal ASD because of small samples. IPV: intimate partner violence, PTSD: post-traumatic stress disorder. NA: no biological plausibility for postnatal psycho social stress to influence infant lung function at 6-10 weeks as both were measured around the same time. FN: few numbers to run the model. Models adjusted for sex, socioeconomic status, race, exposure to benzene, perinatal complications and maternal age, respiratory illness or HIV

**Supplementary table 5. Cross-sectional associations between** **psychosocial stress measures and respiratory resistance or respiratory compliance**

| **Psychosocial stress factors** | **Respiratory resistance (6-10 weeks)** | | | | **Respiratory compliance (6-10 weeks)** | | | |
| --- | --- | --- | --- | --- | --- | --- | --- | --- |
|  | Crude **models (95%CI)** | | **Adjusted models (95%CI)** | | Crude **models (95%CI)** | | **Adjusted models (95%CI)** | |
|  | **β Coefficient (95%CI)** | **p-value** | **β Coefficient (95%CI)** | **p-value** | **β Coefficient (95%CI)** | **p-value** | **β Coefficient (95%CI)** | **p-value** |
| **Prenatal IPV** | -0.035  (-0.109,0.038) | 0.343 | **-0.131 (-0.244,-0.018)** | **0.023** | **-1.8^-4^ (-3.1^-4^,-6.0^-4^)** | **0.004** | -1.9^-4^ (-1.2^-4^,8.3^-5^) | 0.713 |
| **Prenatal PTSD exposure** |  |  |  |  |  |  |  |  |
| None | Ref |  | Ref |  | Ref |  | Ref |  |
| Suspected exposure | 0.020  (-0.043,0.085) | 0.527 | 0.010  (-0.080,0.102) | 0.819 | 2.0^-4^  (-2.3^-4^,6.4^-4^) | 0.369 | -2.9^-5^ (-1.2^-4^,6.2^-5^) | 0.529 |
| Definite exposure | 0.062  (-0.016,0.140) | 0.122 | 0.077  (-0.048,0.202) | 0.226 | 2.7^-4^  (-2.3^-4^,6.4^-4^) | 0.237 | 3.0^-5^ (-1.5^-4^,2.1^-4^) | 0.745 |
| **Prenatal PTSD^a^ exposure** | 0.029  (-0.006,0.065) | 0.109 | 0.033  (-0.023,0.090) | 0.245 | 1.5^-4^  (-1.2^-4^,4.2^-4^) | 0.289 | 5.9^-6^ (-7.0^-5^,8.2^-5^) | 0.880 |
| **Prenatal depression** | -0.009  (-0.047,0.028) | 0.615 | 0.044  (-0.051,0.060) | 0.869 | 2.3^-5^  (-9.7^-5^,5.1^-5^) | 0.539 | -2.4^-5^ (-1.0^-4^,5.4^-5^) | 0.541 |

^a^Assumption of no departure from linear trend for postnatal ASD because of small samples. IPV: intimate partner violence, PTSD: post-traumatic stress disorder. NA: no biological plausibility for postnatal psycho social stress to influence infant lung function at 6-10 weeks as both were measured around the same time. FN: few numbers to run the model. Models adjusted for sex, socioeconomic status, race, exposure to benzene, perinatal complications and maternal age, respiratory illness or HIV

**Supplementary table 6. Interaction of psychosocial adversity with maternal smoking, alcohol consumption and breastfeeding**

|  | **Functional Residual Capacity** | | **Tidal volume** | | **Respiratory rate** | | **t_PTEF_/t_E_** | |
| --- | --- | --- | --- | --- | --- | --- | --- | --- |
|  | **Adjusted β Coefficient (95%CI)** | **p-value** | **Adjusted β Coefficient (95%CI)** | **p-value** | **Adjusted β Coefficient (95%CI)** | **p-value** | **Adjusted β Coefficient (95%CI)** | **p-value** |
| **Prenatal IPV** |  |  |  |  |  |  |  |  |
| Maternal smoking | -0.16  (-0.20,-0.11) | <0.0001 | -0.04  (-0.04,-0.03) | <0.001 | -0.08  (-0.10,-0.06) | <0.0001 | 0.06  (-0.05,0.19) | 0.287 |
| Maternal alcohol use in pregnancy | 0.17  (0.11-0.23) | <0.0001 | 0.06  (0.03,0.10) | <0.001 | -0.14  (-0.15,-0.13) | <0.0001 | -0.06  (-0.28,0.15) | 0.562 |
| Breastfeeds child | 0.005  (-0.004,-0.015) | 0.273 | 0.04  (0.03,0.04) | <0.001 | -0.06  (-0.11,-0.01) | 0.011 | 0.12  (-019,0.44) | 0.459 |
| **Postnatal IPV** |  |  |  |  |  |  |  |  |
| Maternal smoking | -0.14  (-0.22,-0.06) | 0.001 | 0.12  (0.07,0.18) | <0.0001 | -0.19  (-0.26,-0.13) | <0.0001 | -0.26  (-0.42,-0.10) | 0.001 |
| Maternal alcohol use in pregnancy | 0.17  (0.09,0.25) | <0.0001 | 0.03  (0.02,0.04) | <0.0001 | -0.05  (-0.07,-0.04) | <0.0001 | 0.15  (0.01,0.28) | <0.025 |
| Breastfeeds child | -0.03  (-0.07,-0.00) | 0.050 | 0.02  (0.01,0.03) | <0.0001 | 0.05  (-0.04,0.16) | 0.267 | 0.11  (0.08,0.13) | <0.0001 |
| **Prenatal PTSD exposure** |  |  |  |  |  |  |  |  |
| Maternal smoking | -0.22  (-0.63,0.18) | 0.282 | 0.10  (0.09,0.1) | <0.0001 | 0.13 (0.02,0.23) | 0.013 | -0.29  (-0.41,-0.18) | <0.0001 |
| Maternal alcohol use in pregnancy | FN | FN | FN | FN | FN | FN | FN | FN |
| Breastfeeds child | FN | FN | FN | FN | FN | FN | FN | FN |
| **Postnatal PTSD^a^ exposure** |  |  |  |  |  |  |  |  |
| Maternal smoking | FN | FN | FN | FN | FN | FN | FN | FN |
| Maternal alcohol use in pregnancy | FN | FN | FN | FN | FN | FN | FN | FN |
| Breastfeeds child | FN | FN | FN | FN | FN | FN | FN | FN |
| **Prenatal depression** |  |  |  |  |  |  |  |  |
| Maternal smoking | 0.23  (0.23,0.14) | <0.0001 | 0.02  (0.01,0.04) | <0.0001 | 0.14  (0.09,0.18) | <0.0001 | 0.11  (-0.03,0.30) | 0.130 |
| Maternal alcohol use in pregnancy | -0.06  (-0.132,0.00) | 0.041 | FN | FN | FN | FN | FN | FN |
| Breastfeeds child | 0.22  (0.15,0.28) | <0.0001 | FN | FN | FN | FN | FN | FN |
| **Postnatal depression** |  |  |  |  |  |  |  |  |
| Maternal smoking | 0.011  (0.008,-0.014) | <0.0001 | -0.13  (-0.25,-0.01) | 0.027 | 0.08  (0.05,0.10) | <0.0001 | 0.028  (0.006,0.050) | 0.010 |
| Maternal alcohol use in pregnancy | FN | FN | FN | FN | FN | FN | FN | FN |
| Breastfeeds child | FN | FN | FN | FN | FN | FN | FN | FN |

Models adjusted for sex, socioeconomic status, race, exposure to benzene, perinatal complications and maternal age, respiratory illness or HIV and with interaction terms between psychosocial adversity and maternal smoking, alcohol consumption and breastfeeding. Interaction results reported for the most significant of the different log levels of psychosocial adversity. Maternal smoking was an ordered variable (no, smoking, passive smoking or active smoking); maternal alcohol consumption was a binary variable (no smoking or smokes); breastfeeds child was a binary variable (no breastfeeding or breastfeeds). FN: too few numbers to run the interaction analysis.
