# Supplementary figures and images for: Association between maternal psychological adversity and lung function in South African infants: A birth cohort study
Source: Pediatr Pulmonol. 2019 Sep 30;55(1):236–44. doi: 10.1002/ppul.24532 (PMC7154702; doi:10.1002/ppul.24532)

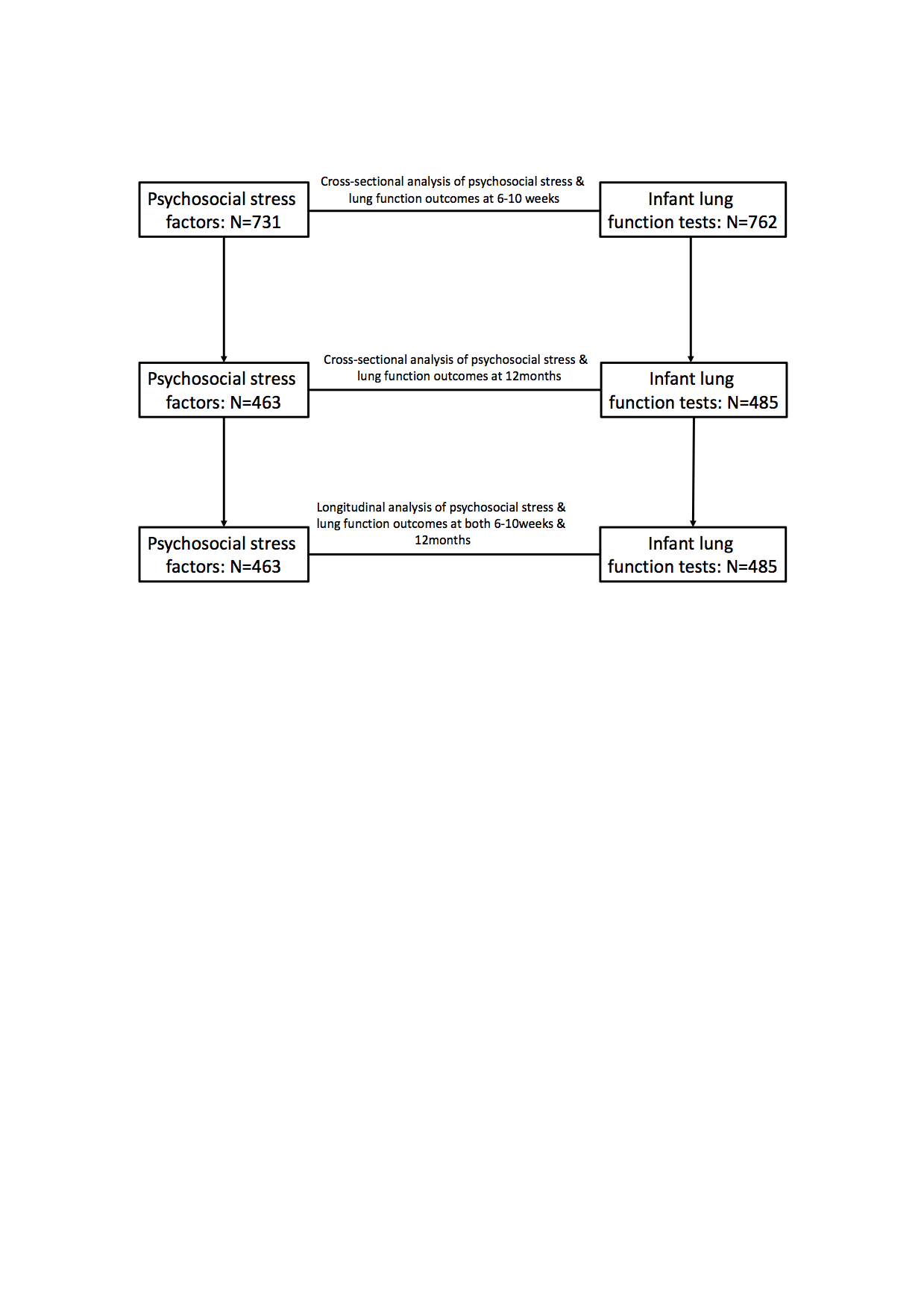

Supplement: Supplementary file 2 — Supporting information [file PPUL-55-236-s002.tiff]
